# Next-generation sequencing analysis reveals high bacterial diversity in wild venomous and non-venomous snakes from India.

**DOI:** 10.1186/s40409-018-0181-8

**Published:** 2018-12-22

**Authors:** Sajesh Puthenpurackal Krishnankutty, Megha Muraleedharan, Rajadurai Chinnasamy Perumal, Saju Michael, Jubina Benny, Bipin Balan, Pramod Kumar, Jishnu Manazhi, Bangaruswamy Dhinoth Kumar, Sam Santhosh, George Thomas, Ravi Gupta, Arun Zachariah

**Affiliations:** 1 AgriGenome Labs Pvt. Ltd., SmartCity Kochi, Kakkanad, Kerala 682042 India; 2 Department of Forests and Wildlife, Sulthan Batheri, Wayanad District, Kerala 673592 India; 3 SciGenom Research Foundation, Cheruthuruthy, Kerala 679531 India; 4 Medgenome Labs Pvt. Ltd., Narayana Health City, Bommasandra, Bengaluru, Karnataka 560099 India

**Keywords:** Microbial community, Next-generation sequencing, Venomous snake, Hypervariable region

## Abstract

**Background:**

The oral cavities of snakes are replete with various types of bacterial flora. Culture-dependent studies suggest that some of the bacterial species are responsible for secondary bacterial infection associated with snakebite. A complete profile of the ophidian oral bacterial community has been unreported until now. Therefore, in the present study, we determined the complete bacterial compositions in the oral cavity of some snakes from India.

**Methods:**

Total DNA was isolated from oral swabs collected from three wild snake species (Indian Cobra, King Cobra and Indian Python). Next, the DNA was subjected to PCR amplification of microbial 16S rRNA gene using V3-region-specific primers. The amplicons were used for preparation of DNA libraries that were sequenced on an Illumina MiSeq platform.

**Results:**

The cluster-based taxonomy analysis revealed that Proteobacteria and Actinobacteria were the most predominant phyla present in the oral cavities of snakes. This result indicates that snakes show more similarities to birds than mammals as to their oral bacterial communities. Furthermore, our study reports all the unique and common bacterial species (total: 147) found among the oral microbes of snakes studied, while the majority of commonly abundant species were pathogens or opportunistic pathogens to humans. A wide difference in ophidian oral bacterial flora suggests variation by individual, species and geographical region.

**Conclusion:**

The present study would provide a foundation for further research on snakes to recognize the potential drugs/antibiotics for the different infectious diseases.

**Electronic supplementary material:**

The online version of this article (10.1186/s40409-018-0181-8) contains supplementary material, which is available to authorized users.

## Background

Vertebrates form mutual relationships with huge and complex microbial flora that inhabit their gastrointestinal tract. A major proportion of these microbes probably assist in essential processes of energy and nutrient acquisition in the host [1]. The combination of next-generation DNA sequencing methods, ecological aspects and bioinformatics analysis tools is rapidly expanding our comprehension of the evolution and function of vertebrate-related bacterial communities [2, 3]. The diet and genotype impact the bacterial diversity, since the bacterial communities co-diversified with their hosts [4]. Most of the studies have tended to characterize fecal microbiomes from captive animals, often from laboratories or zoos [1]. However, captive microbial community likely do not represent the natural variation of the microbiome of a species (or population), which is necessary for evolutionary analysis [5]. Most studies investigating evolutionary patterns in vertebrate gut microbiomes have focused on mammals and birds only [6, 7]. Until now, very few studies have analyzed the gut microbiome of squamate reptiles (snakes and lizards) despite this being one of the most diverse and successful vertebrate clades [2, 8].

Presently, the use of reptiles has increased in the investigations of infectious disease, comparative anatomical physiology, stem cell experiments, evaluation of phylogenic relations with birds and other vertebrates, and therapeutic drug development [9–13]. Among the reptiles, snakes have been utilized for the isolation of different types of peptides from venom for numerous purposes. Microorganisms, including bacteria and fungi, naturally inhabit the oral cavity and gut of snakes [14–17]. The literature suggests that oral cavities of venomous and non-venomous snakes are colonized by numerous species of anaerobic and aerobic bacteria [18, 19]. Since the ophidian oral bacteria may be inoculated during a snake’s bite, bacterial multiplication and infection may occur under favorable conditions. A strong connection has been established between microorganisms present in abscesses or in patients’ lesions and those from snakes’ oral cavities [20].

Snakebite-generated secondary wound infections involve a polymicrobial mixture of microorganisms originating from the ophidian oral cavity. Bites from non-venomous snakes can also cause injury, as a result of lacerations by the snake’s teeth, and subsequent infection [21]. The identification of snake-associated microorganisms is imperative to extend our insight into these life forms that inhabit the buccal cavity, and furthermore to acquire understanding of the etiological operators of secondary infections resulting from accidents during handling. Hence, the profile of microbial vulnerability to antimicrobials must be investigated to encourage the development of adequate treatments of human accidents and snake bacterial infections.

It has been suggested that oral microbiota of snakes reflects the fecal flora of their ingested preys since these sufferers frequently defecate at the moment they are being ingested [22]. However, recent culture-independent high-throughput sequencing studies identified that bacterial taxa present in the oral cavity of snakes were distinct from fecal microbiota of their prey [23]. Despite the influence of associations of bacteria and snakes and the influence of these bacteria on humans, there are a few studies on the characterization and distribution of these microorganisms [24, 25]. Next-generation deep sequencing of hypervariable regions from 16S ribosomal RNA genes is a useful tool for understanding the microbial communities in several organisms [26]. Recently, a metagenomic sequencing study on the Timber Rattlesnake has unveiled the complete gut microbiome that is essential for the health and nutrition of the species, and the microorganisms associated with disease transmission between this snake and other animals [27]. However, complete snake oral metagenomic sequencing has not been reported until now. Therefore, the objective of the present study is to identify the bacterial community diversity in the oral cavity of two venomous and one non-venomous species of snake native to India.

## Methods

### Microbial sampling

**Fig. 1:**
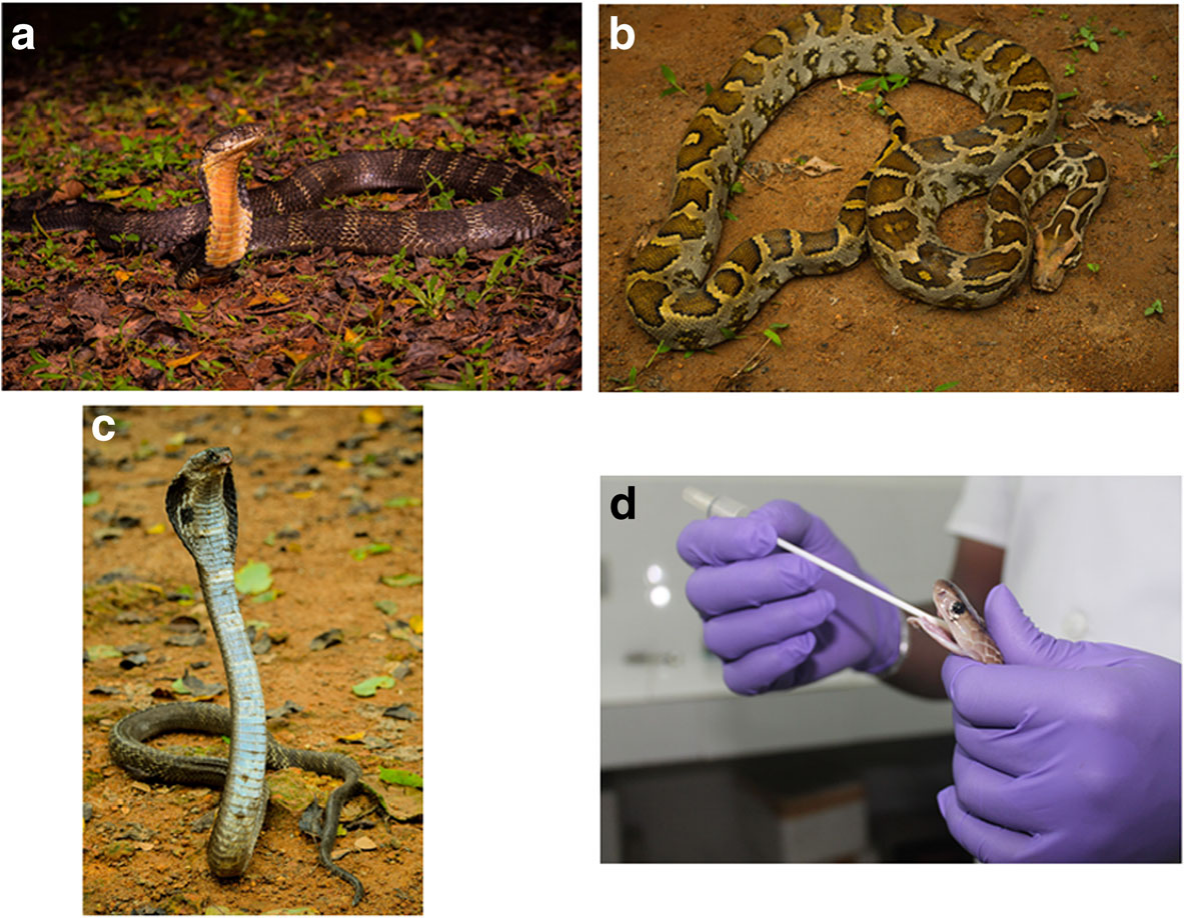
Photographs of venomous and non-venomous species of snakes used for the oral sample collection. **a** King Cobra (*Ophiophagus hannah*); **b** Indian Python (*Python molurus*); **c** Indian Cobra *(Naja naja*); **d** Oral sample collection from a snake using commercially available sterile cotton-tipped swab stick

According to the availability, a total of four snakes from three species were used in this study. Live venomous snakes, namely one Indian Cobra *(Naja naja*) and one King Cobra (*Ophiophagus hannah*), and a non-venomous Indian Python (*Python molurus*) were captured from the wild from the Wayanad district of Kerala state, India (Fig. 1a, b and c). The snakes were handled carefully throughout the exercise with the help of snake handlers/experts. Upon capture, each snake was transported immediately to the laboratory. Soon after the arrival of snakes, oral samples were collected. The animal handler secured the head while a veterinarian opened the mouth of the snake using a sterile wooden spatula. Oral swab samples were collected from each snake using commercially available sterile cotton-tipped swab sticks (Fig. 1d). After collection, swabs were placed separately into sterile tubes and transported immediately to the laboratory on ice. Snakes were released back into the wild after the exercise. A fresh road-killed King Cobra presented in a veterinary hospital near the laboratory was also used for the sample collection.

### DNA isolation, amplification, and sequencing

Total DNA was isolated from oral swab using the QuickExtract™ DNA Extraction Solution (Epicentre, USA) as per the manufacturer’s instructions. The isolated DNA was checked for purity using the spectrophotometer NanoDrop™ 2000 (ThermoFisher Scientific, USA) and quantified with Qubit 2.0 Fluorometer (Invitrogen, USA). The DNA was stored at − 20^0^ C until further use.

Library preparation of samples was employed using the kit Nextera XT Index (Illumina, USA) as per the manufacturer’s protocol. The steps involved firstly the PCR amplification of microbial 16S rRNA gene fragments using V3 region specific primers 341F-5’CCTACGGGAGGCAGCAG3’ and 534R-5’ATTACCGCGGCTGCTGG3’ with 25 μl reaction containing 2 μl each 10 pmol μl − 1 forward and reverse primers, 0.5 μl of 40 mM dNTP, 5 μl of New England Biolabs® 5× Phusion HF reaction buffer, 0.2 μl of 2 U μl^− 1^ F-540 Special Phusion HS DNA Polymerase, and 10 ng DNA. The PCR cycling conditions included an initial denaturation at 98 °C for 30 s followed by 30 cycles of 98 °C for 10 s, 72 °C for 30 s, extension at 72 °C for 5 s followed by a final hold at 4 °C employing the thermal cycler ABI (ThermoFisher Scientific, USA). The amplicon DNA was further purified by using PureLinkTM Quick Gel Extraction (Invitrogen, USA) and visualized with SYBR® Safe DNA gel stain (10 μl/100 ml). The second phase PCR was performed after tagging the library with universal primers and Illumina indexed bar code sequences. The PCR Master Mix contained 2 μL each of 10 pmol/ul forward and reverse primers, 1 μL of 40 mM dNTP, 10 μL of 5 X Phusion HF reaction buffers, 0.4 μl of 2 U/μl F-540 Special Phusion HS DNA Polymerase, 10 μl (minimum 5 ng) of amplicon from the previous PCR cycle and water to complete the total volume of 50 μl. The final library products were validated on a Tape Station 2200 instrument (Agilent Technologies, USA) using the Agilent 2200 Tape Station software. The library was then loaded on Illumina MiSeq platform with a 300-cycle Illumina MiSeq reagent kit v.2 for achieving paired-end sequencing (2 × 150 bp paired end run). The raw FASTQ file data of four samples; Cobra, King Cobra 1 (KC1), the road-killed King Cobra 2 (KC2) and Python were deposited in NCBI’s Sequence Read Archive under BioProject ID: PRJNA408014 under the respective Biosample accession numbers SRR6053311, SRR6053312, SRR6053313 and SRR6053314.

### Taxonomy profiling and community analysis of 16S rRNA amplicon sequences

Initially, the sample raw sequencing reads were checked for quality, adapter dimer and duplication using FastQC V0.11.5, whereas the adapter trimming was performed using an in-house PERL script. The sequences with Phred score ≥ 30 (>Q30; error-probability > = 0.001) were considered for further downstream analysis. The adapter trimmed reads were merged to make V3 consensus FASTA using the FLASH program with default parameters. All the chimeric sequences were detected and filtered using the UCHIME algorithm as the de novo chimera-removal method. The pre-processed reads were clustered into Operational Taxonomic Units (OTU) using Uclust proGram with the similarity cutoff of 0.97. The singleton OTUs (read abundance < 2) were discarded from the analysis. The data was then analyzed using the software package QIIME V1.8 (Quantitative Insights into Microbial Ecology) to reveal and elucidate the taxonomy profile of samples. The representative sequence was picked for each of the OTUs and mapped against Greengenes and SILVA core set Small sub-unit (SSU) reference database using PyNAST proGram. Taxonomy from phylum to species level was assigned to each OTU representative sequence with the RDP classifier using a confidence threshold of 0.8. The taxon diversity study (richness and evenness) within the samples was performed employing Shannon, Chao1, whereas the observed species metrics calculation and diversity between samples were accomplished via distance matrix calculation and principal component analysis (PCA). The OTU network maps were generated using QIIME and visualized with Cytoscape [28].

### Statistical analysis

Phylogenetic Investigation of Communities by Reconstruction of Unobserved States (PICRUST) analysis was employed to study the functional gene profile of metabolic pathways among the samples. The metabolic profile was further analyzed using the software package Statistical Analysis of Metagenomic Profiles (STAMP) v2.0. The statistically significant *P*-values were calculated based on Fisher’s exact test method using Storey’s false discovery rate method of multiple test correction within STAMP, considering *P*-values *< 0.05* for comparison.

## Results

### Sequence analysis

The next-generation sequencing of partial 16S rRNA genes based on taxonomy profiling employed in this study inferred the bacterial diversity in the oral cavities of three different Indian snake species, namely the Indian Cobra, King Cobra and Indian Python. Oral swabs from the snakes were collected and the V3 hypervariable region of 16S rRNA gene of microbiome was sequenced by the Illumina-based method. Total readings of 1,155,180, 1,085,952, 1,065,656, 1,404,982 were obtained for Cobra, King Cobra (KC1), fresh road-killed King Cobra (KC2) and Python samples, respectively. The average GC content of all the samples were 52–54% and an average base quality Phred score of 93–97% (Table 1). After pre-processing, the V3 sequences for each sample were generated. The sequences obtained from each sample were first pooled together and then clustered using the program Uclust, available in QIIME V1.8 with similarity cutoff of 0.97. From a total of 46,907 OTUs, 24,233 singleton OTUs (<=1 read) were removed and 22,674 OTUs were considered for further analysis.

**Table 1:** Data and analysis summary of snake oral samples

Category	Cobra	King Cobra 1	King Cobra 2	Python
Number of Reads (R1 + R2)	1,155,180	1,085,952	1,065,656	1,404,982
Average Phred Score	35.85	35.35	36.01	35.96
Average GC (%)	52.51	56.41	53.68	55.56
Total V3 sequences	417,478	355,250	395,623	515,755
Chimeric Filters	369	3140	1755	21,147
Total OTUs	870	4506	2510	20,709

### Taxonomic profiling of metagenomic sequences

**Fig. 2:**
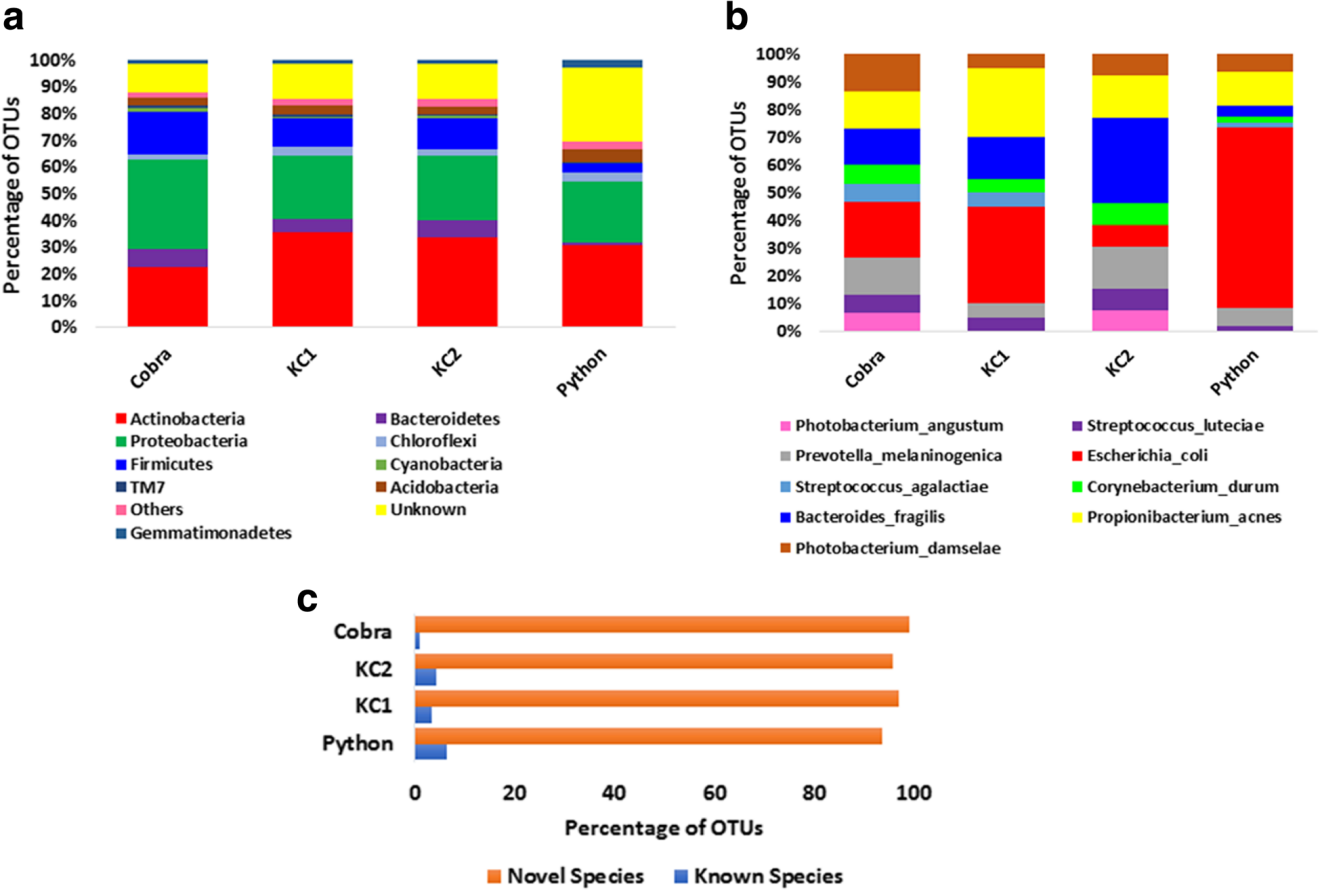
Taxonomy relative abundance plot of Cobra, King Cobra and Python oral samples. **a** The bar plot represents the relative OTU abundance of Cobra, King Cobra 1 (KC1), King Cobra 2 (KC2) and Python samples at the phylum level. In total, about 88% of OTUs were assigned to a known phylum while 12% of OTUs were designated as unknown. Actinobacteria and Proteobacteria were predominantly present in all the samples; **b** The bar plot shows percentage-wise relative OTU abundance at the species level. *Escherichia coli*, *Bacteroides fragilis* and *Propionibacterium acnes* were the most dominant species among the samples; **c** The plot shows the percentage of known and novel species identified after OUT-based clustering and annotation. Forty percent of the total OTUs were classified into a known genus. Likewise, 10% of OTUs were assigned a known species taxonomy classification. Here: novel species = unclassified or unknown species, known species = taxonomy information is available in the database

**Fig. 3:**
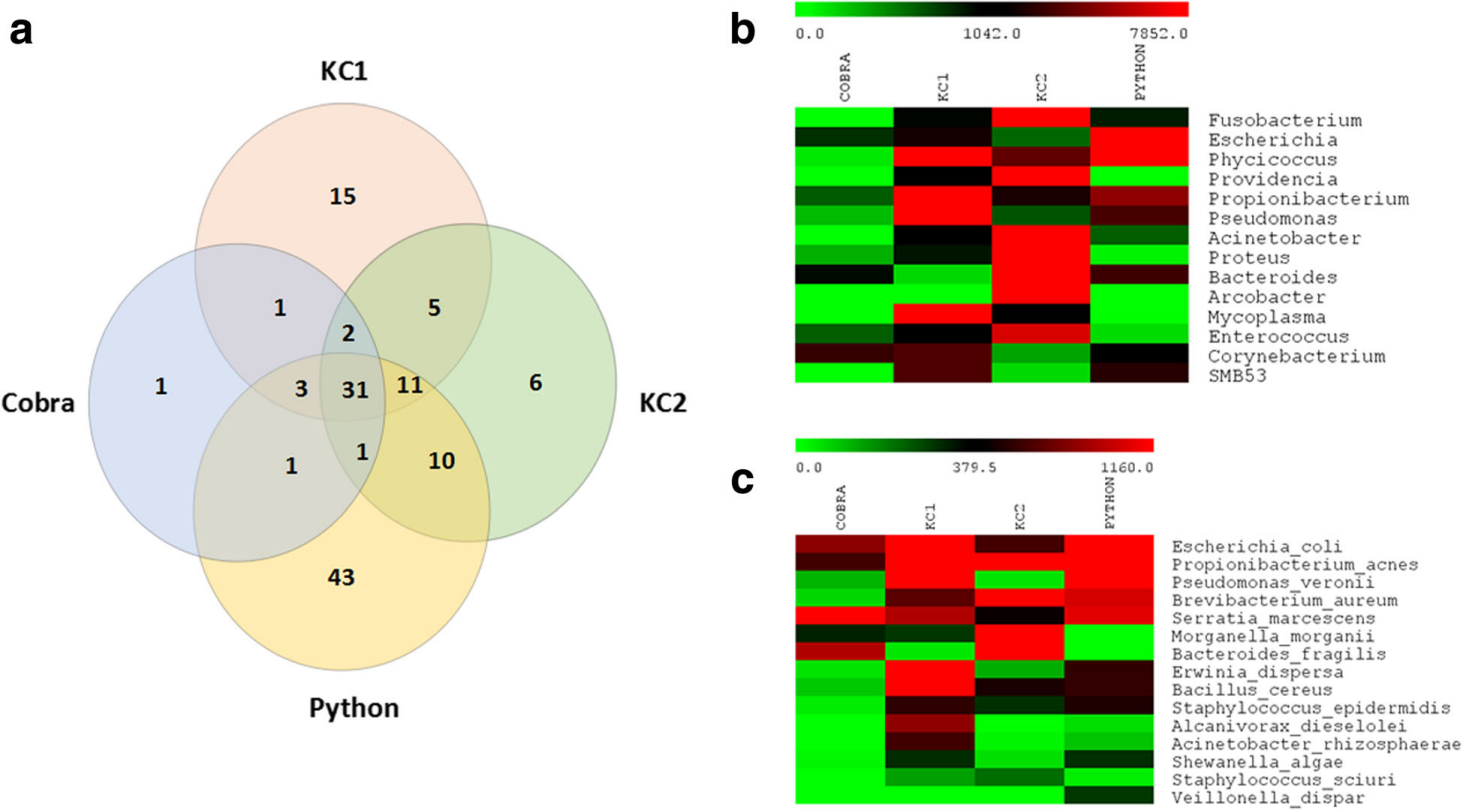
Venn diagram and heat-map representation of bacterial species identified among snake oral samples. **a** Venn diagram shows the number of common and uniquely identified species. There are 31 bacterial species commonly present (out of total 147 bacterial species) among Python, Cobra, King Cobra 1 and King Cobra 2; **b** Heat map indicates the most abundant bacterial genera among these snakes; **c** Heat map shows top 15 commonly abundant bacterial species in descending order

The taxonomic classification of OTUs was carried out using RDP classifier against Greengenes and SILVA 16S RNA gene database [29, 30]. The relative distribution of phyla, genera and species between the samples is shown in Fig. 2. The nine phyla – including Actinobacteria, Bacteroidetes, Proteobacteria, Chloroflexi (Chlorobacteria), Firmicutes, Cyanobacteria, TM7 (Candidatus Saccharibacteria), Acidobacteria and Gemmatimonadetes – were commonly distributed among the samples (Fig. 2a). Our results demonstrated that Proteobacteria (Cobra: 33.4%, KC1: 23.5%, KC2: 24.3%, Python: 22.8%) and Actinobacteria (Cobra: 22.8%, KC1: 36.01%, KC2: 33.8%, Python: 30.7%) were identified as the most predominant phyla associated with the snakes analyzed. At the species level, according to the OTU-based relative taxon abundance, *Photobacterium angustum*, *Streptococcus luteciae*, *Prevotella melaninogenica*, *Escherichia coli*, *Streptococcus agalactiae*, *Corynebacterium durum*, *Bacteroides fragilis*, *Propionibacterium acnes* and *Photobacterium damselae* were found among all the samples (Fig. 2b). The complete taxonomy annotation summary is displayed as Additional file 1. The sequences that did not associate with any known reference taxon were classified as unknown or novel hits (Fig. 2c). A total of 96% of unique OTUs are reported as unknown at the species level, since there were no hits. Of the 22,674 total OTUs, we identified 147 unique species among all the four samples. The distribution of common and unique species between the samples is shown in Fig. 3a. A total of 31 species were shared by all the four samples, 43 species were unique to Python, 15 to KC1, 6 to KC2 and only one species was uniquely present in the Cobra sample. The unique species present in the Cobra oral cavity was *Bifidobacterium adolescentis.* The heat map indicates that most reads matched to *Corynbacterium* being the most abundant at the genus level, followed by *Baceroides* and *Escherichia* in the Cobra, *Phycicoccus, Propionibacterium, Pseudomonas* and *Mycobacterium* in KC1, *Fusobacterium*, *Providencia*, *Acinetobacter*, *Proteus* and *Baceroides* in KC2, and *Escherichia coli* and *Phycicoccus* in the Python (Fig. 3b). By combining data from all snakes, it was found that *Escherichia coli, Propionibacterium acnes*, *Pseudomonas veronii, Brevibacterium aureum, Serratia marcescens* and *Morganella morganii* were the most abundant bacteria at the species level (Fig. 3c).

Taxonomic analysis indicated that 50, 93, 76 and 125 bacterial species in the oral cavities of Cobra, KC1, KC2 and Python, respectively, and the majority of commonly abundant species were pathogens or opportunistic pathogens to humans. The top ten abundant bacterial species are listed in Table 2. *Serratia marcescens* was most abundant followed by *Bacteroides fragilis*, *Escherichia coli* and *Propionibacterium acnes* in the Cobra snake. In KC1, *Peudomonas veronii* and *Propionibacterium acnes* were the most abundant followed by *Erwinia dispersa* and *Escherichia coli*. In descending order of their abundance, the oral cavity of KC2 was occupied by *Morganella morganii*, *Brevibacterium aureum*, *Bacteroides fragilis* and *Propionibacterium acnes*. Among the known species of the bacterial community, *Escherichia coli* was highly present and together with *Propionibacterium acnes*, *Pseudomonas veronii*, *Serratia marcescens* and *Brevibacterium aureum*, it contributed the greatest share of the bacterial species in the Python.

**Table 2:** Top ten bacterial species present in oral cavities of three snake species in India

SI No	Cobra	King Cobra 1	King Cobra 2	Python
1	*Serratia marcescens (1678)*	*Pseudomonas veronii (7938)*	*Morganella morganii (2937)*	*Escherichia coli (46438)*
2	*Bacteroides fragilis (917)*	*Propionibacterium acnes (7246)*	*Brevibacterium aureum (2645)*	*Propionibacterium acnes (4634)*
3	*Escherichia coli (807)*	*Erwinia dispersa (2303)*	*Bacteroides fragilis (2528)*	*Pseudomonas veronii (1160)*
4	*Propionibacterium acnes (592)*	*Escherichia coli (1931)*	*Propionibacterium acnes (1713)*	*Serratia marcescens (1061)*
5	*Morganella morganii (321)*	*Bacillus cereus (1159)*	*Eubacterium dolichum (1688)*	*Brevibacterium aureum (1025)*
6	*Pseudomonas veronii (115)*	*Serratia marcescens (911)*	*Escherichia coli (605)*	*Bacillus cereus (554)*
7	*Bacillus cereus (85)*	*Alcanivorax dieselolei (817)*	*Bacillus cereus (481)*	*Erwinia dispersa (549)*
8	*Brevibacterium aureum (66)*	*Brevibacterium aureum (667)*	*Parabacteroides gordonii (458)*	*Staphylococcus epidermidis (495)*
9	*Erwinia dispersa (49)*	*Acinetobacter rhizosphaerae (595)*	*Serratia marcescens (438)*	*Shewanella algae (301)*
10	*Staphylococcus epidermidis* *(37)*	*Staphylococcus epidermidis* *(543)*	*Staphylococcus epidermidis* *(302)*	*Veillonella dispar* *(290)*

**Fig. 4:**
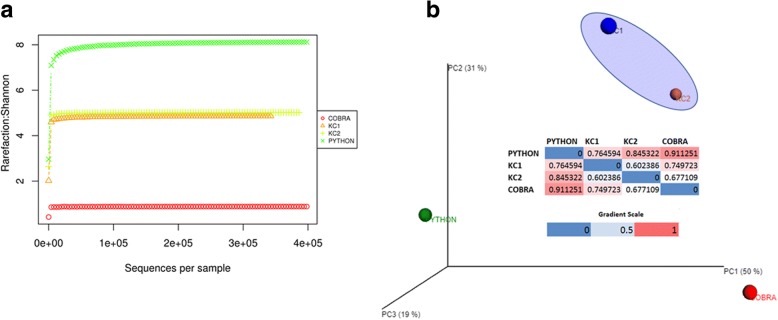
Alpha and beta diversity analysis of snake oral samples. **a** The rarefaction curve plotted using Shannon metrics to observe the species richness and evenness within the samples. The Shannon diversity index, estimated as 8.12 based on observed OTU abundances, indicates that the Python oral cavity (green asterisk) contains a higher number of species than the other samples; **b** Principal Coordinate Analysis of bacterial communities among the samples. The plot indicates that the King Cobra 1 and King Cobra 2 samples share a common taxon. Python and Cobra were uniquely distributed. All metrics were calculated using the software QIIME v1.8

The rarefaction plots elucidate that the Python has more taxa (i.e., common: 31 and unique: 43) in the oral cavity as compared to the King Cobra (KC1 and KC2) and Cobra (Fig. 4a). The alpha diversity result is shown in Additional file 2. PCA analysis revealed that the King Cobra samples (KC1 and KC2) clustered closely by sharing identical OTUs at the phylum and species level, whereas bacterial species in the Python and Cobra were uniquely distributed and qualitatively deviated from KC1 and KC2 (Fig. 4b).

### Comparison of bacterial community structure and statistical difference between the snakes

**Fig. 5:**
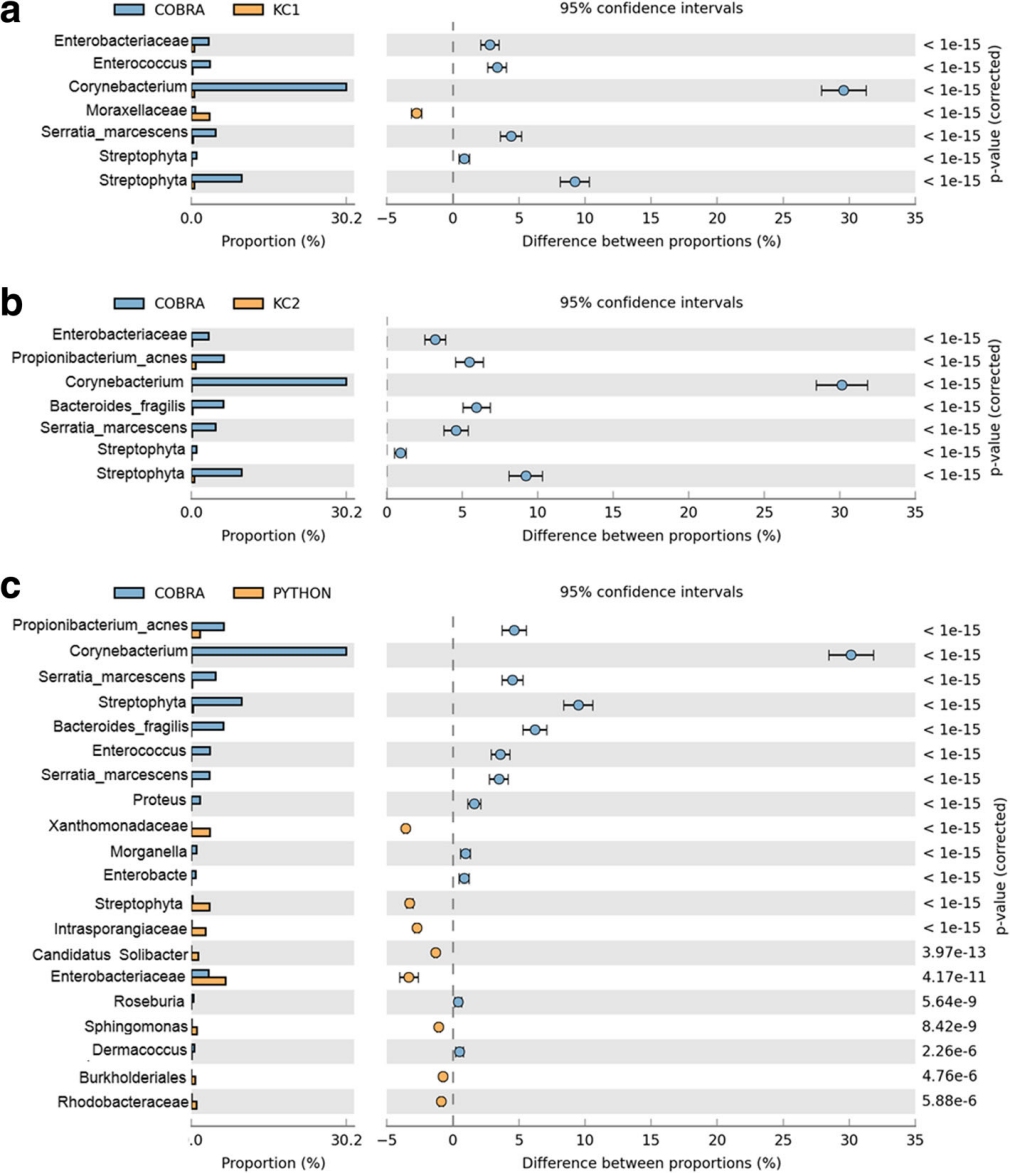
Metagenomic profile comparisons of Python, Cobra and King Cobra oral samples determined using STAMP analysis. The comparison includes highly significant phylum to species level. Corrected *P*-values were calculated based on Fisher’s exact test method using Storey’s FDR approach. *P*-values < 0.05 were taken for comparison. The bar plot indicated in blue or orange shows a positive or negative difference between read proportions. Differences between samples are shown at 95% confidence intervals **a** Taxon comparison between Cobra and KC1 samples. *Corynebacterium* is present in greater abundance in Cobra and lesser abundance in KC1 with positive differences (blue dot), whereas *Moraxellaceae* is less abundant in Cobra and more abundant in KC1 with negative differences (yellow dot); **b** Comparison of Cobra and KC2. The most abundant taxon includes *Corynebacterium, Bacteroides fragilis* and *streptophyta*, all with positive proportion differences; **c** Comparison between Cobra and Python samples. *Corynebacterium, Propionibacterium acnes* and *serratia marcescens* are highly abundant with positive differences, whereas the species group including Xanthomonadaceae, Streptophyta and Enterobacteriaceae are in greater abundance with negative differences. Here, KC1: King Cobra 1 and KC2: King Cobra 2

The comparison of taxa using STAMP V1.2 [31] shows that *Enterobacteriacea*, *Corynebacterium*, *Enterococcus*, *Streptococcus* and *Xanthomonadaceae* were significantly overrepresented with positive difference (*P* < 1e-15) in the 16S rRNA gene amplicon surveys of Cobra, KC1, KC2 and Python. However, *Moraxellaceae*, *Propionibacterium acnes* and *Serratia marcescens* were overrepresented with negative proportion differences (Fig. 5). The *P*-values were estimated based on Fisher’s exact test method using Storey’s FDR approach. The correlations between Cobra and King Cobra shows that dominant microbes were positively correlated with *P* < 1e-15 significance. However, comparison of the Cobra with the Python revealed significant negative correlations in the abundance (*P* < 1e-6) of Enterobacteriacea, Xanthomonadaceae and Streptophya.

## Discussion

Very few studies limited to study of gut microbiota have attempted to characterize the bacterial organisms colonizing the snake gastrointestinal tract. Except for some culture-dependent studies, a complete profile of the oral bacterial community was absent in this species [32, 33]. Herein, we investigated the oral bacterial community composition of venomous and non-venomous snakes using 16S rRNA amplicon sequencing analysis.

The present study demonstrated that, within the phylum level, Proteobacteria and Actinobacteria were dominant in the oral bacterial community of the snakes studied. In partial 16S rRNA Illumina sequencing of captive Komodo dragon, one of the reptilian species’ oral data showed that Bacteroidetes and Firmicutes shared top abundance of 27.9 and 28.6%, followed by Proteobacteria (18.9%) and Actinobacteria (13%) [8]. The researchers also reported that microbiota present in the oral cavity and skin of the Komodo dragon are similar to those in its environment, but less equivalent to the stool-associated microbiota. However, there has been no phylum-level sequencing evidence yet reported for a snake oral microbiome. 16S amplicon sequencing of the gut microbiota of the Timber Rattlesnake (*Crotalus horridus*) showed that Proteobacteria population dominated in the small intestine and colon (85%), whereas, inside the stomach, Proteobacteria and Firmicutes were predominant at 50 and 40%, respectively [27]. The pyrosequencing of the Cottonmouth snake (*Agkistrodon piscivorus*) gastrointestinal tract has revealed that the large intestine, small intestine and cloaca were dominated by sequences associated with Proteobacteria, Bacteroidetes and Firmicutes [2]. Compared to mammalian oral microbiota, where bacteria from the phyla Bacteroidetes, Firmicutes and Proteobacteria typically dominate [34–36], the oral microbiota in our snake species were characterized by expanded abundances of Actinobacteria and Proteobacteria. A recent study demonstrated a dominant level of Actinobacteria and Proteobacteria in the oral cavity of a free-living passerine bird, the Great Tit (*Parus major*) [37]. The dominance of these two bacterial phyla in the oral cavities of snakes in our study suggests that snakes may show more similarities to birds as to their oral bacterial communities than to other vertebrate organisms.

Like other creatures, snakes’ oral cavity is a suitable place for bacterial growth and some of them represent normal oral flora of animals in general. Studies on oral bacterial flora in snakes have been undertaken worldwide using culture-based methods [19, 38]. Different bacterial species have been recognized from the oral cavity of various varieties of snakes. The most significant ones are *Pseudomonas* and *Aeromonas* [39], *Morganella morganii* [40], *Staphylococcus aureus*, *Escherichia coli*, *Proteus*, *Colestridia*, *Enterococcus*, coagulase-negative *Staphylococcus* [41], S*tenotrophomonas maltophilia* [42], *Acinetobacter*, *Klebsiella*, and *Shigella* [43, 44], *Staphylococcus*, *Salmonella*, *Escherichia* and *Providencia* [32]. In our current study, the greatest number of bacterial species were found in the Python specimens (a total of 125) followed by King Cobra. When compared to other snakes, the Cobra oral cavity contained the lowest number of bacterial species. The bacterial community in all the snakes was observed to be mixed population of Gram-positive and Gram-negative organisms, and the commonly abundant bacteria were pathogens or opportunistic pathogens to humans. The wide spectrum of pathogens found in the oral cavity of the venomous snakes studied, whose bites may cause not only poisoning but also infection, aggravates the condition in victims. The literature indicates that bites from non-venomous snakes may also result in secondary bacterial infection [19]. In this study, *Propionibacterium acnes*, S*erratia marcescens* and *Erwinia dispersa* were the commonly found pathogenic species in the non-venomous Python. Subsequently, people associated with snake transportation or studies using these animals run the risk of being infected by opportunistic pathogens. The likelihood of infection is particular high for persons who are sick or immunocompromised.

Recently, Shaikh and co-workers isolated a total of 205 bacterial strains from the oropharyngeal cavity of four snake species including the Indian Cobra, Russell’s viper, Saw-scaled viper, and Common Krait [33]. These bacterial species mainly comprise *Morganella morganii*, *Escherichia coli*, *Aeromonas hydrophila*, *Pseudomonas aeruginosa*, coagulase-negative *Staphylococcus aureus*, Bacillus spp., Micrococcus spp., and some anaerobes including *Clostridium perfringens*. In the present study, we found 50 bacterial species in the oral flora of the Indian Cobra including an extensive variety of Gram-negative bacteria mainly constituted by *Serratia marcescens, Bacteroides fragilis, Escherichia coli* and *Morganella morganii,* but also by *Propionibacterium acnes* and *Bacillus cereus,* the commonest Gram-positive bacteria. Earlier, a group of workers detailed more than 50 bacterial species in the oral flora of the Chinese Cobra including Aeromonas, Proteus, Colestridium spp., *Staphylococcus aureus*, Enterococcus, and coagulase-negative Staphylococcus [38]. Previous reports in literature similar to this study showed the presence of *Serratia marcescens* [15, 45]*, Bacteroides fragilis* [38, 46], *Escherichia coli* [41, 47], *Morganella morganii* [40] and *Propionibacterium acnes* [22] in the oral cavity of snakes. We also found the presence of some of the soil bacteria like *Bacillus cereus* in the oral cavities of the snakes studied. Frequent flicking of the tongue together with feeding and drinking may inoculate the buccal cavity with these bacteria.

In this current study, the Python possessed the greatest number of bacterial species with *E.coli* was identified as the most common followed by *Propionibacterium acnes*, *Pseudomonas veronii* and *Serratia marcescens*. Oral samples from free-living Reticulated Pythons presented high prevalence of *Staphylococcus sciuri, Acinetobacter genomospecies, Aeromonas hydrophila* and *Pseudomonas aeruginosa* [48]. Pythons – usually found in grasslands, swamps, marshes, rocky foothills, woodlands and river valleys – depend on a source of water [49]. Unlike other snake species, Pythons typically consume a correspondingly large variety of prey such as frogs, fishes, small lizards, earthworms, aquatic insects etc., due to their body size, to gain the energy required for capture, ingestion and digestion. Thus, the wide range of bacterial species present in the Python oral cavity might be due to its varied range of habitats and foods. Although both the King Cobra samples used in this study showed similarity in the oral flora at the phylum level, there were wide variations at the genus and species level. The oral cavities of KC1 and KC2 contained 93 and 76 bacterial species, respectively. We demonstrated that among the all the snakes studied, 15 species were unique to KC1 and 6 were exclusively present in KC2. These results were corroborated by the previous report that snakes of the same species do not necessarily harbor the same bacterial flora and numbers [19]. The king cobra KC2 was a fresh road-killed one and therefore, death might be another reason for the difference in numbers of bacterial species between KC1 and KC2.

Snakebite is a serious and important issue in tropical and subtropical countries. It primarily brings the consequences of envenomation and can cause a lesion at the bite site with extensive necrosis. The dead tissue can secondarily get infected by bacteria coming from the snake’s mouth that might be inoculated at instance of the bite [50]. Mixed bacterial infections were commonly observed in wound cultures with a combination of Gram-positive, Gram-negative, and anaerobic microorganisms. In Taiwan, snake (*Trimeresurus mucrosquamatus*, *Trimeresurus stejnegeri* and Cobra) wound cultures demonstrated a high prevalence of *Morganella morganii* and *Enterococcus* spp. [51]. A later study in Taiwan also reported high abundance of *Morganella morganii* in a snakebite wound [52]. Other commonly found species have been *Enterococcus* spp., *Proteus* spp., *Aeromonas hydrophila*, *Pseudomonas aeruginosa*, and *Providencia* spp. A recent bacteriological analysis of snakebite wound from South Africa also showed *Morganella morganii* was the most predominant bacteria followed by *Proteus* spp. [53]. In agreement with other studies, the present manuscript reported a prevalence of *Morganella morganii* in all the three species studied. Earlier, researchers from India reported that *Staphylococcus aureus* (32%) was the most common isolate followed by *Escherichia coli* (15%) in the snakebite wound infection [54].

Our current study has demonstrated that ophidian oral cavities were predominantly occupied by Gram-negative bacteria including *Escherichia coli*, *Pseudomonas veronii*, *Serratia marcescens*, *Morganella morganii*, *Bacteroides fragilis* and *Erwinia dispersa*. These Gram-negative bacteria have the ability to cause serious health complications in the host once the victims are exposed to snakebite attacks. Absorption and dissemination of endotoxins of Gram-negative bacteria by the blood can be accompanied by severe clinical symptoms such as low blood fibrinogen level, hypotension, acute shock and death [55]. The common Gram-positive bacterial species found in the oral cavities of snakes used in the present study were *Propionibacterium acnes*, *Brevibacterium aureum*, *Bacillus cereus*, *Eubacterium dolichum* and *Staphylococcus epidermidis. Propionibacterium acnes* is a pathogenic bacterium, whereas *Bacillus cereus* and *Staphylococcus epidermidis* are part of normal human microflora and behave as opportunistic pathogens. Studies have already demonstrated the antibacterial activity of snake venom [56–58]. The results suggest that the presence of antibacterial molecules in the snake venom would protect the snakes during feeding. In the current study, the non-venomous Python possessed more numerous bacterial species than venomous snakes. The lower number of bacteria found in the Cobra oral cavity may be due to the process of envenomation. Future studies on more snake varieties from different geographical regions of India are warranted to enable a detailed comparative analyses to investigate the origin and diversity of oral-cavity-associated bacterial communities.

## Conclusions

In this study, for the first time, we have shown oral bacterial flora in the venomous and non-venomous snake species from India using next-generation sequencing of hypervariable regions from 16S rRNA gene. Our study demonstrated a wide variation in bacterial species among these snakes whose oral cavities were predominantly occupied by both Gram-negative and Gram-positive, pathogenic or opportunistic pathogenic bacteria. Our finding of a wide difference in ophidian oral bacterial flora suggests variation by individual, species and geographical region. The results generated from this study are of concern, as a bite inflicted by these snakes can result in wound infections and tissue necrosis leading to sepsis/necrotizing fasciitis and/or expose snake handlers, veterinarians and researchers to infections. Furthermore, this work provides a foundation to carry out further research on snakes to recognize the potential drugs/antibiotics for treating different infectious diseases.

## Additional files

Additional file 1: The taxonomy annotation and the relative abundance all the samples from phylum to species level. (XLS 158 kb)

Additional file 2: The alpha diversity metrices of all samples calculated based on Shannon Index method. The index values shows the species richness and evenness of the sample. (XLS 123 kb)

## Abbreviations

KC: King cobra
OTU: Operational taxonomic units
PCA: Principal component analysis
PICRUST: Phylogenetic Investigation of Communities by Reconstruction of Unobserved States
rRNA: Ribosomal RNA
STAMP: Statistical analysis of metagenomic profiles
